# Characterization of Uropathogenic *Escherichia coli* Reveals Hybrid Isolates of Uropathogenic and Diarrheagenic (UPEC/DEC) *E. coli*

**DOI:** 10.3390/microorganisms10030645

**Published:** 2022-03-17

**Authors:** Rodrigo H. S. Tanabe, Regiane C. B. Dias, Henrique Orsi, Daiany R. P. de Lira, Melissa A. Vieira, Luís F. dos Santos, Adriano M. Ferreira, Vera L. M. Rall, Alessandro L. Mondelli, Tânia A. T. Gomes, Carlos H. Camargo, Rodrigo T. Hernandes

**Affiliations:** 1 Departamento de Ciências Químicas e Biológicas (Setor de Microbiologia e Imunologia), Instituto de Biociências, Universidade Estadual Paulista (UNESP), Botucatu 18618-689, SP, Brazil; rodrigohtanabe@outlook.com (R.H.S.T.); reg.chrys@hotmail.com (R.C.B.D.); henrique.orsi@unesp.br (H.O.); daiany.lira@unesp.br (D.R.P.d.L.); melissa.vieira@unesp.br (M.A.V.); vera.rall@unesp.br (V.L.M.R.); 2 Centro de Bacteriologia, Instituto Adolfo Lutz, São Paulo 01246-902, SP, Brazil; luis.santos@ial.sp.gov.br (L.F.d.S.); carlos.camargo@ial.sp.gov.br (C.H.C.); 3 Hospital das Clínicas da Faculdade de Medicina de Botucatu, Botucatu 18607-741, SP, Brazil; adrianomartison@hotmail.com; 4 Departamento de Clínica Médica, Faculdade de Medicina, Universidade Estadual Paulista (UNESP), Botucatu 18618-970, SP, Brazil; alessandro.mondelli@unesp.br; 5 Departamento de Microbiologia, Imunologia e Parasitologia, Universidade Federal de São Paulo-Escola Paulista de Medicina (UNIFESP-EPM), São Paulo 04023-062, SP, Brazil; tatg.amaral@unifesp.br

**Keywords:** UPEC, DEC, hybrid *E. coli*, virulence, antimicrobial resistance, ESBL

## Abstract

(1) Background: Pathogenic *Escherichia coli* are divided into two groups: diarrheagenic (DEC) and extraintestinal pathogenic (ExPEC) *E. coli*. ExPEC causing urinary tract infections (UTIs) are termed uropathogenic *E. coli* (UPEC) and are the most common cause of UTIs worldwide. (2) Methods: Here, we characterized 112 UPEC in terms of phylogroup, serotype, the presence of virulence factor-encoding genes, and antimicrobial resistance. (3) Results: The majority of the isolates were assigned into the phylogroup B2 (41.07%), and the serogroups O6 (12.5%) and O25 (8.9%) were the most frequent. Five hybrid UPEC (4.5%), with markers from two DEC pathotypes, i.e., atypical enteropathogenic (aEPEC) and enteroaggregative (EAEC) *E. coli*, were identified, and designated UPEC/aEPEC (one isolate) and UPEC/EAEC (four isolates), respectively. Three UPEC/EAEC harbored genes from the *pap* operon, and the UPEC/aEPEC carried *ibeA*. The highest resistance rates were observed for ampicillin (46.4%) and trimethoprim/sulfamethoxazole (34.8%), while 99.1% of the isolates were susceptible to nitrofurantoin and/or fosfomycin. Moreover, 9.8% of the isolates were identified as Extended Spectrum β-Lactamase producers, including one hybrid UPEC/EAEC. (4) Conclusion: Our data reinforce that hybrid UPEC/DEC are circulating in the city of Botucatu, Brazil, as uropathogens. However, how and whether these combinations of genes influence their pathogenicity is a question that remains to be elucidated.

## 1. Introduction

*Escherichia coli*, a member of the family *Enterobacteriaceae*, is generally a harmless commensal of the gastrointestinal tract [1]. However, there are several *E. coli* clones that have acquired the ability to produce virulence factors, which provides them with the potential to cause several infectious diseases in humans and animals [2,3]. Genes encoding virulence factors associated with the *E. coli* pathogenesis are often located in mobile genetic elements, such as plasmid, phage, and pathogenic island (PAI), that can be mobilized into different *E. coli* backgrounds, thus originating many distinct genetic profiles [4,5,6,7,8,9].

Pathogenic *E. coli* are divided into two major groups: diarrheagenic (DEC) and extraintestinal pathogenic *E. coli* (ExPEC), which causes diarrhea and extraintestinal infections, such as neonatal meningitis, sepsis, and urinary tract infections (UTIs), respectively [2,3,10]. Based mainly on the virulence repertory and phenotypic features, DEC is currently classified into six distinct pathotypes [2]. Atypical enteropathogenic (aEPEC), which differs from typical EPEC (tEPEC) by the absence of the EPEC adherence factor (EAF) plasmid [11], and enteroaggregative (EAEC) *E. coli* are the most common DEC pathotypes isolated from diarrheal patients in Brazil [12,13] as well as worldwide [14], even though aEPEC and EAEC have also been frequently isolated from healthy subjects with asymptomatic intestinal infections [13,15,16,17]. In addition, *E. coli* isolates harboring EPEC and EAEC markers have also been implicated as etiologic agents of extraintestinal infections, i.e., UTIs and bloodstream infections [18,19,20,21,22].

*E. coli* isolates causing UTIs are collectively known as uropathogenic *E. coli* (UPEC) and are the most common cause of this infection in both out- and inpatients worldwide [23]. Virulence strategies of UPEC include adherence to urinary tract epithelial cells, iron acquisition, toxins production, and evasion of the host’s immune system [24,25]. These virulence factors are frequently encoded by genes located in pathogenicity islands (PAIs), most often acquired by horizontal gene transfer [6,26,27].

Phylogenomic analyses have consistently demonstrated that the *E. coli* species is very complex and structured in eight distinct phylogroups, as follows: A, B1, B2, C, D, E, F, and the newly described G [28,29,30]. The large majority of the UPEC isolates have been assigned into phylogroup B2, in addition to isolates observed in other *E. coli* phylogroups [31,32].

Mainly since the mid-2000s, the constant increase of UPEC rates with resistance to several antimicrobial drugs has been undermining the effectiveness of clinical management of UTIs, thus becoming a worldwide public health concern [33]. Of particular importance we can highlight the UPEC isolates that produce extended-spectrum beta-lactamases (ESBL) enzymes and/or are multidrug-resistant (MDR) [34,35].

The main goal of this study was to characterize a collection of UPEC isolates obtained from outpatients of the Hospital of the Medical School of Botucatu, Brazil, regarding their serotype, presence of virulence factor-encoding genes, and antimicrobial resistance profiles. In addition, all the isolates were assigned in the distinct *E. coli* phylogroups.

## 2. Material and Methods

### 2.1. UPEC Isolates

This study analyzed 112 UPEC isolates obtained from outpatient urine samples with counts ≥10^5^ colony-forming units (CFU) per mL of urine. The 112 outpatients were attended to at the Clinical Hospital from the Medical School–University of São Paulo State (UNESP)–Botucatu, São Paulo State, Brazil, between March, April, and May 2018.

### 2.2. Virulence Factor-Encoding Genes Detection

The presence of a total of 29 genes encoding proteins associated with distinct roles in the pathogenesis of ExPEC isolates, such as adhesins (*fimH*, *ecpA, papA*, *papC*, *iha, afaBC*, *sfa/focDE*, *hra,* and *bamE*), toxins (*usp*, *sat*, *vat*, *hlyA*, *hlyF*, *picU*, *cnf1*, *tsh,* and *cdt*), iron acquition (*iroN*, *irp2*, *iucD*, *ireA,* and *sitA*), serum resistance (*tratT*, *ompT*, *iss*, *cva,* and *kpsMTII*) and the invasion-encoding gene *ibeA*, were investigated in the UPEC isolates by DNA amplification. Polymerase Chain Reaction (PCR) was performed using Green Master Mix (Promega, Madison, WI, USA) with 0.34 μM of each primer. All primer sequences and PCR conditions used to detect virulence genes are described in the references cited in Table S1.

Moreover, genes that are frequently used for the diagnosis of some DEC pathotypes, such as EPEC (*eae* and *bfpB*), EAEC (*aggR* and *aatA*), and STEC (*stx1/2*), were investigated using primers described in Table S2. Positive and negative controls were included in all PCR reactions as described in previous publications from our laboratory [17,36].

### 2.3. Serotyping

The identification of somatic (O) and flagellar (H) antigens was performed by standard agglutination tests [37], with specific O1–O181 and H1–H56 antisera produced at the Instituto Adolfo Lutz, São Paulo, Brazil [12].

### 2.4. E. coli Phylogroup Classification

The UPEC isolates were assigned into the distinct *E. coli* phylogroups (A, B1, B2, C, D, E, and F) and clades using a quadruplex PCR [28]. Subsequently, UPEC isolates classified in the phylogroup B2, with the following genotype: *arpA*^−^, *chuA*^+^, *yjaA*^−^, and TspE4^+^, and all UPEC isolates from the phylogroup F were submitted to a triplex PCR [29] to confirm these isolates as B2 and F, or to reclassify them into the newly described phylogroup G.

### 2.5. Antimicrobial Resistance Profile and Detection of ESBL-Producing E. coli

Antimicrobial susceptibility assays were performed using the disk diffusion method, performed on Mueller-Hinton agar (OXOID, Basingstoke, UK). The inhibition zone diameter was interpreted following the Clinical and Laboratory Standards Institute [38], except for cephalothin, a first-generation cephalosporin, whose breakpoint was from a previous publication [39].

The 19 antimicrobial drugs tested were: ampicillin (AMP; 10 μg), piperacillin/tazobactam (PPT, 100/10 μg), ampicillin/sulbactam (ASB, 10/10 μg), amoxicillin/clavulanic acid (AMC; 20/10 μg), cephalotin (CFL, 30 μg), cefuroxime (CRX, 30 μg), ceftazidime (CAZ, 30 μg), cefotaxime (CTX, 30 μg), cefepime (CPM, 30 μg), ertapenem (ETP, 10 μg), meropenem (MER, 10 μg), gentamicin (GEN, 10 μg), amikacin (AMI, 30 μg), nalidixic acid (NAL, 30 μg), norfloxacin (NOR, 10 μg), ciprofloxacin (CIP, 5 μg), trimethoprim/sulfamethoxazole (SUT, 1.25/23.75 μg), nitrofurantoin (NIT, 300 μg), and fosfomycin (FOS, 200 μg). All commercial disks impregnated with antimicrobial drugs tested were obtained from Cefar Diagnóstica Ltd. (São Paulo, SP, Brazil), and the *E. coli* ATCC 25922 was used as quality control from the disks. Multidrug-resistant (MDR) *E. coli* was defined by the detecting of non-susceptible isolates (resistant or intermediate) to at least one agent of three or more of the six distinct classes of antimicrobial drugs tested, as follows: (1) β-lactams, (2) aminoglycosides, (3) quinolones/fluoroquinolones, (4) folic acid metabolism inhibitors, (5) nitrofuran, and (6) phosphonic acid derivative [40,41].

Extended-spectrum β-lactamase (ESBL) production was investigated using ceftazidime (30 μg/mL) and cefotaxime (30 μg/mL) with and without clavulanic-acid (10 μg/mL). An increase of 5 mm or more in the zone diameter, observed in the presence of clavulanic acid, was considered positive for ESBL production [38].

### 2.6. Detection of Beta-Lactamase-Encoding Genes

Genes encoding the main ESBL groups, such as TEM (*bla_TEM-1_*), SHV (*bla_SHV_*), and CTX-M (*bla_CTX-M-2-group_*, *bla_CTX-M-8-group_*, and *bla_CTX-M-15-group_*), as well as the AmpC cephalosporinase CMY-2 (*bla_CMY-2_*), were investigated by PCR using primers as described in Table S3.

## 3. Results

The large majority of outpatients from which the 112 UPEC isolates were obtained was female (90.2%, 101/112), with ages ranging from 18 to 65 years (65.3%, 66/101) (Table S4). Clinically, the outpatients with community acquired UTIs were diagnosed as follows: cystitis (77.7%, 87/112), pyelonephritis (9.8%, 11/112), or asymptomatic bacteriuria (9.8%, 11/112). In addition, three outpatients (2.7%) did not have their diagnosis documented in their medical records (Table S4).

Regarding somatic antigen (O) typing, which defines the *E. coli* serogroups, 64 UPEC isolates (57.1%, 64/112) were identified in 31 distinct serogroups, with the serogroups O6 (12.5%, 14/112) and O25 (8.9%, 10/112) as the most frequently detected, besides the occurrence of O-non-typeable (28.6%, 32/112) or autoagglutinable (14.3%, 16/112) UPEC isolates (Table S5). Considering the O:H serotypes, O6:H1 and O25:H4 were the most frequent and equally detected in 7.1% (8/112) of the UPEC isolates studied (Table S5).

We observed that a large number of isolates were assigned into the *E. coli* phylogroup B2 (41.07%, 46/112), followed by isolates assigned in the phylogroups A (16.07%, 18/112), B1 (14.29%, 16/112), D (12.5%, 14/112), F (7.14%, 8/112), and G (3.57%, 4/112). Less commonly, we observed UPEC isolates assigned into the phylogroups C, E and *E*. clades, with a frequency of 1.79% (2/112) in each of the phylogroups (Table 1).

Of the 29 virulence factor-encoding genes investigated, the most frequently detected were *fimH* (98.2%, 110/112), *traT* (81.3%, 91/112), *ecpA* (77.7%, 87/112), *ompT* (62.5%, 70/112), *irp2* (52.7%, 59/112), and *sitA* (51.8%, 58/112) (Table 1). Some particularities should be highlighted, such as the occurrence of some genes in just one or two phylogroups, such as *sfa/focDE*, *cnf1,* and *cdt,* exclusively detected in isolates from the phylogroup B2; *ibeA* only in isolates from the phylogroups B2 and F; and *vat* only in isolates from the phylogroups B2 and G (Table 1). UPEC with the highest number of virulence factor-encoding genes were observed in isolates assigned to phylogroup B2, with a mean of 9.5, containing UPEC isolates harboring a minimum of three to a maximum of 17 genes (Table 1). A high mean of virulence factor-encoding genes was also observed in the phylogroups D (8.3), G (7.8), and F (6.1) (Table 1).

Five UPEC isolates (4.5%) harbored virulence markers frequently used for diagnosis of two important DEC pathotypes, aEPEC (*eae*^+^) and EAEC (*aggR*^+^ and *aatA*^+^), and thus were designated as hybrid UPEC/aEPEC (one isolate) and UPEC/EAEC (four isolates), respectively (Table 2). The five UPEC/DEC hybrid isolates were obtained from four patients diagnosed with cystitis and one with pyelonephritis; the patients were of both genders (four female and one male), aged from 1 to 84 years (Table 3). Other relevant clinical information from the patients from which the hybrid UPEC/DEC isolates were obtained is summarized in Table 3. All five patients who had UTIs due to hybrid UPEC/DEC isolates progressed to the curing of the infection (data not shown).

These hybrid isolates were heterogeneous in terms of serotype, phylogroup classification and content of genes encoding virulence factors associated with the ExPEC pathogenesis, as shown in Table 2. All five hybrid isolates harbored the *fimH*, *ecpA*, *traT,* and *ompT* genes, three UPEC/EAEC isolates (75%, 3/4) harbored genes from the *pap* operon (*papA* and/or *papC*) and/or *irp2*, while the *ibeA* gene was observed only in the UPEC/aEPEC isolate from the phylogroup B2 (Table 2).

The highest resistance rates were observed for the following antimicrobial drugs: ampicillin (46.4% 52/112) and trimethoprim/sulfamethoxazole (34.8%, 39/112), as well as for the quinolones/fluoroquinolones tested, as follows: nalidixic acid (31.3%, 35/112), ciprofloxacin (31.3%; 35/112) and norfloxacin (20.5%, 23/112) (Table 4). On the other hand, 99.1% (111/112) of the UPEC isolates tested were susceptible to nitrofurantoin and/or fosfomycin (Table 4). Thirty-one isolates (27.7%, 31/112), including two hybrid UPEC/EAEC (isolates 85 and 100), were classified as MDR, with the majority of them (77.4%, 24/31) showing non-susceptibility to the following classes of antibiotics tested concomitantly: β-lactams, quinolones/fluoroquinolones and folic acid metabolism inhibitors (Table S6). Worryingly, 9.82% (11/112) were classified as an ESBL-producing UPEC (Table 5). The majority (72.73%, 8/11) harbored the *bla_CTX-M-15-group_*, with this gene detected with the AmpC cephalosporinase-encoding *bla_CMY-2_* gene in two isolates concomitantly (Table 5).

Furthermore, an overview of the resistance profile of the five hybrid isolates identified in this study (four UPEC/EAEC and one UPEC/aEPEC) is summarized in Table 6. We observed that the UPEC/EAEC hybrid isolates were non-susceptible to antimicrobial drugs of the following classes: β-lactams (100.0%, 4/4), aminoglycosides (50.0%, 2/4), quinolones/fluoroquinolones (50.0%, 2/4), and folic acid metabolism inhibitors (50.0%, 2/4). On the other hand, the hybrid UPEC/aEPEC isolate 92 was susceptible to all antimicrobial drugs tested (Table 6). Of note, one hybrid UPEC/EAEC (isolate 85) was identified as an ESBL-producer (Table 6).

## 4. Discussion

In the present study, we provide an extensive molecular and phenotypic characterization of Brazilian UPEC isolates, such as the classification of UPEC isolates in the distinct *E. coli* phylogroups, the presence of virulence factor encoding-genes, O:H serotypes, and antimicrobial resistance profiles. Together, we observed that UPEC isolates circulating in the city of Botucatu are very heterogeneous in all features investigated, similar to what was observed in other studies carried out in Brazil, as well as in other countries [31,32,42,43]. Despite the high diversity observed, some characteristics seem to be common when comparing UPEC isolates from distinct geographic regions, such as the majority of the isolates being assigned to phylogroup B2 and serotype O25:H4 as one of the most commonly detected [31,32].

DEC and ExPEC isolates use distinct virulence factors to cause intestinal and extraintestinal diseases in the human host, respectively [2,3]. However, the identification of hybrid *E. coli* isolates, combining virulence factor encoding-genes from both pathogenic *E. coli* groups, i.e., DEC and ExPEC, is becoming more and more frequent in the literature [18,20,21,44,45,46,47]. Among the most common DEC molecular markers that have been identified in UPEC, we can highlight the locus of enterocyte effacement (LEE region), a chromosomal PAI from EPEC, and the aggregative adherence plasmid (pAA), from EAEC [18,20,45,47]. Less frequently, UPEC isolates harboring the *stx1* and/or *stx2* genes, which are molecular markers of Shiga toxin-producing *E. coli* (STEC) that encode potent cytotoxins that inhibit protein synthesis in eukaryotic cells [48], have also been detected [46,49,50].

The LEE encodes proteins that induce the formation of a histopathological lesion, termed attaching and effacement (AE), in infected epithelial cells [51]. The AE lesion is characterized by intimate adherence of bacteria to the host cell, which is mediated by the interaction between the adhesin intimin and its translocated receptor, microvillus effacement, F-actin accumulation, and the formation of a pedestal-like structure underneath adherent bacteria [52,53]. A recent study has compiled evidence that a hybrid aEPEC/UPEC-252 isolate (serotype O71:H40) harbor all 41 genes from the LEE region, produces the adhesin intimin (encoded by the *eae* gene located in the LEE region), adheres to both urinary (T24 and HEK 293T) and intestinal (Caco-2 and LS174T) cell lineages, and induces AE lesions in HeLa cells [54]. The latter study may be the first to demonstrate that the LEE region, present in *E. coli* isolates obtained from extraintestinal sites, can be functional. However, whether the proteins encoded by genes from the LEE region contribute to the establishment, or aggravation, of UTIs has yet to be determined.

The hybrid UPEC/aEPEC-92 (UPEC isolate harboring the LEE region isolated in this study) belongs to serotype O145:H34 and harbored the *ibeA* gene. This gene was first identified in a meningitis-associated *E. coli* (MNEC) K1 as part of the GimA (genetic island of meningitis *E. coli* containing *ibeA*) PAI, which is organized in four distinct operons (*ptnIPKC*, *cglDTEC*, *gcxKRCI,* and *ibeRAT*). Of note, IbeA contributes to MNEC K1 crossing the blood-brain barrier [55]. Even though this isolate was first classified as UPEC, due to the site of isolation, and then reclassified as a hybrid UEPC/aEPEC, due to the presence of the LEE region, the presence of the *ibeA* gene from MNEC cannot be ignored. This type of data brings to light all the complexity in the pathogenic *E. coli* classification, which is based on the infection site for ExPEC isolates, and in the presence of molecular markers for DEC isolates [56]. In Brazil, aEPEC of serotype O145:H34 has also been isolated from diarrheal patients [12] and, similar to the hybrid UPEC/aEPEC-92, belongs to the phylogroup B2 [57,58]. Together, these data suggest that UPEC and/or aEPEC of serotype O145:H34 may act as a heteropathogen, which can be defined as pathogenic *E. coli* isolates that occupy an evolutionary interface between ExPEC and DEC (hybrid ExPEC/DEC isolates), thus, being able to cause disease in the intestinal and extraintestinal sites of human hosts [45,49].

Another important genetic element from DEC that has been frequently found in UPEC isolates is the pAA [18,21,44,47], which is characteristic of EAEC isolates. EAEC is defined as *E. coli* isolates that produce the aggregative adherence pattern (AA) on and around epithelial cells, characterized by a bacterial arrangement that resembles stacked bricks [59]. The pAA harbors an operon that encodes the proteins necessary for the biogenesis of one of the five AAF (aggregative adherence fimbria) types (AAF/I–AAF/V), which is responsible for the establishment of the AA pattern [60,61]; besides, it carries the genes encoding other EAEC-associated virulence factors, such as a type I secretion system (*aatPABCD*), the anti-aggregation protein dispersin (*aap*), heat-stable enterotoxin EAST1 (*astA*), plasmid-encoded toxin (*pet*), shigella extracellular protein (*sepA*), as well as a transcriptional activator of virulence genes (*aggR*) [62]. Studies from the literature have shown that some hybrid UPEC/EAEC isolates can produce the AA pattern on HeLa cells, as well as form biofilm on abiotic surfaces [18,20,45,47], thus demonstrating that some hybrid UPEC/EAEC isolates preserve the ability to produce phenotypes compatible with isolates from the EAEC pathotype.

The pathogenic potential of UPEC/EAEC isolates can be illustrated by an UTI outbreak that occurred in Copenhagen, Denmark, during the year 1991, due to an *E. coli* strain of serotype O78:H10 that harbored several EAEC virulence-associated genes, such as *sat*, *pic*, *aatA*, *aggR*, *aggA*, *aaiC,* and *aap* [19]. An additional study demonstrated that C555-91 (a representative isolate from the Copenhagen UTI outbreak) causes urovirulence in a mouse model without the requirement for AAF/I. In addition, biofilm formation in urethral catheters is dependent on this fimbria production [63], thus indicating at which stage of the hybrid UPEC/EAEC infection the EAEC virulence factors are really necessary. There is also a report in the literature of a 55-year-old female patient hospitalized with persistent diarrhea and vomiting who had a UTI followed by sepsis [64]. In this patient, EAEC of serotype O176:HNT was isolated from urine and blood samples, and three types of DEC were isolated from the stool, two of which were EAEC (serotypes O176:HNT and O12:H4) and one aEPEC (O56:H6). Although it seems evident that EAEC of serotype O176:HNT caused the UTI and sepsis, the isolation of three types of DEC from the stool sample makes it challenging to identify the pathogen associated with the diarrheal disease and, consequently, the association of the EAEC O176:HNT as a heteropathogen. By associating the uropathogenic potential of these *E. coli* isolates (serotypes O78:H10 and O176:HNT) with the presence of EAEC markers, we can extrapolate the information from the studies mentioned above and conclude that these isolates are, in fact, hybrid UPEC/EAEC. Furthermore, EAEC with uropathogenic features are present in the stool of asymptomatic individuals and may be a source of UTI [65].

Isolates of *E. coli* serotype O3:H2, molecularly classified as EAEC, have been frequently isolated from stool samples from patients with diarrhea, as well as from asymptomatic individuals in studies carried out in Brazil [12,66,67,68,69]. In the present study, the hybrid UPEC/EAEC-34 of serotype O3:H2 was isolated from a UTI, thus suggesting that some bacteria of this serotype may act as a heteropathogen. Except for serotype O3:H2, the other serotypes identified among the UPEC/EAEC isolates in this study (O15:H6, O126:H10, and O90:H2) seem not to be associated with cases of diarrhea caused by EAEC in the Brazilian setting [12,68].

Usually, community-acquired UTI is more responsive to antimicrobial treatment and has lower antimicrobial resistance rates than hospital-acquired UTI [70]. In Brazil, the current consensus recommends the use of fosfomycin and nitrofurantoin as the first choices for the treatment of uncomplicated UTI in women [71], and we found that only one isolate was not susceptible to these drugs in vitro; therefore, empirical recommendations are potentially effective against UPEC in our region.

On the other hand, we detected isolates presenting the MDR phenotype, including ESBL-producing UPEC. We identified CTX-M-8 and CTX-M-15-producing isolates belonging to diversified phylogroups and serotypes, likely indicating multiple acquisitions of these determinants in different evolutionary events. CTX-M-producing *E. coli* with different genetic backgrounds was already reported in clinical UPEC [34] and in environmental *E. coli* isolates from the food chain in Brazil [72]. In addition, an increase in the prevalence of ESBL-producing or quinolone-resistant UPEC was reported in Rio de Janeiro [35], indicating that the occurrence of drug- resistant UPEC is disseminated in different regions. Although quinolones and 3rd-generation cephalosporin are not recommended to treat uncomplicated UTI, alternative drugs are needed for other complicated infections, which can represent a potential threat in terms of resistance dissemination and public health implications [35].

Data on the resistance profile of hybrid UPEC/EAEC and UPEC/aEPEC isolates are scarce in the literature. However, a very recently published article characterizing UPEC isolates obtained from 100 ambulatory patients with community-acquired UTIs in the city of Chilpancingo, Mexico, reported that 77.27% (17/22) of the UPEC/EAEC hybrid isolates studied were classified as ESBL producers, besides the high resistance rates observed for β-lactams, aminoglycosides, quinolones/fluoroquinolones, and folic acid metabolism inhibitors [73]. Moreover, a hybrid UPEC/aEPEC identified as an ESBL-producer, and harboring the *bla_CTX-M-15_* gene, was reported as a causative agent of UTI in Brazil [47].

## 5. Conclusions

Our data reinforce that hybrid UPEC/EAEC and UPEC/aEPEC isolates are circulating in the city of Botucatu, Brazil, as important uropathogens. However, how and whether these specific combinations of genes can influence their pathogenicity is a question that remains to be investigated.

## Figures and Tables

**Table 1:** Occurrence of virulence factor-encoding genes in 112 uropathogenic *E. coli* (UPEC) isolates identified in the distinct *E. coli* phylogroups.

Genes Investigated ^a^	No. (%) of UPEC Isolates Identified in the Distinct Phylogroups									
	A (*n* = 18)	B1 (*n* = 16)	B2 (*n* = 46)	C (*n* = 2)	D (*n* = 14)	E (*n* = 2)	F (*n* = 8)	G (*n* = 4)	*E*. Clades (*n* = 2)	Total (*n* = 112)
Adhesins										
*fimH*	16 (88.9)	16 (100)	46 (100)	2 (100)	14 (100)	2 (100)	8 (100)	4 (100)	2 (100)	110 (98.2)
*ecpA*	11 (61.1)	14 (87.5)	36 (78.3)	2 (100)	12 (85.7)	2 (100)	6 (75)	4 (100)	0	87 (77.7)
*papC*	1 (5.6)	2 (12.5)	18 (39.1)	0	10 (71.4)	0	2 (25)	1 (25)	1 (50)	35 (31.3)
*iha*	2 (11.1)	1 (6.3)	13 (28.3)	0	8 (57.1)	0	1 (12.5)	1 (25)	0 (0)	26 (23.2)
*papA*	1 (5.6)	1 (6.3)	10 (21.7)	0	8 (57.1)	0	3 (37.5)	1 (25)	1 (50)	25 (22.3)
*sfa/focDE*	0	0	19 (41.3)	0	0	0	0	0	0	19 (17)
*afaBC*	1 (5.6)	0	3 (6.5)	0	0	0	0	1 (25)	0	5 (4.5)
*hra*	0	0	0	0	1 (7.1)	0	1 (12.5)	0	0	2 (1.8)
Invasin										
*ibeA*	0	0	16 (34.8)	0	0	0	1 (12.5)	0	0	17 (15.2)
Toxins										
*usp*	0	0	33 (71.7)	0	1 (7.1)	0	2 (25)	0	0	36 (32.1)
*sat*	1 (5.6)	1 (6.3)	11 (23.9)	0	9 (64.3)	0	3 (37.5)	1 (25)	0	26 (23.2)
*vat*	0	0	24 (52.2)	0	0	0	0	1 (25)	0	25 (22.3)
*hlyA*	1 (5.6)	0	8 (17.4)	0	1 (7.1)	0	0	0	0	10 (8.9)
*picU*	1 (5.6)	0	6 (13)	0	1 (7.1)	0	1 (12.5)	1 (25)	0	10 (8.9)
*hlyF*	0	3 (18.8)	3 (6.5)	1 (50)	0	0	0	1 (25)	0	8 (7.1)
*cnf1*	0	0	6 (13)	0	0	0	0	0	0	6 (5.4)
*cdt*	0	0	2 (4.3)	0	0	0	0	0	0	2 (1.8)
Iron Acquisition										
*irp2*	4 (22.2)	3 (18.8)	35 (76.1)	1 (50)	9 (64.3)	0	3 (37.5)	4 (100)	0	59 (52.7)
*sitA*	4 (22.2)	5 (31.3)	34 (73.9)	2 (100)	9 (64.3)	0	3 (37.5)	1 (25)	0	58 (51.8)
*iucD*	1 (5.6)	7 (43.8)	20 (43.5)	1 (50)	6 (42.9)	0	4 (50)	2 (50)	0	41 (36.6)
*ireA*	0	0	8 (17.4)	0	1 (7.1)	0	0	1 (25)	0	10 (8.9)
*iroN*	0	1 (6.3)	4 (8.7)	0	0	0	0	0	0	5 (4.5)
Serum Resistance										
*traT*	11 (61.1)	13 (81.3)	38 (82.6)	2 (100)	14 (100)	2 (100)	6 (75)	4 (100)	1 (50)	91 (81.3)
*ompT*	6 (33.3)	7 (43.8)	38 (82.6)	1 (50)	11 (78.6)	1 (50)	4 (50)	2 (50)	0	70 (62.5)
*iss*	1 (5.6)	2 (12.5)	5 (10.9)	2 (100)	0	0	0	0	0	10 (8.9)
*cva*	0	3 (18.8)	1 (2.2)	0	0	0	0	1 (25)	0	5 (4.5)
*kpsMTII*	0	0	2 (4.3)	0	1 (7.1)	0	1 (12.5)	0	0	4 (3.6)
Range of VFs	0–11	2–9	3–17	6–8	4–12	3–4	2–11	5–11	2–3	0–17
Mean of VFs	3.4	4.9	9.5	7.0	8.3	3.5	6.1	7.8	2.5	7.2

^a^ The *tsh* and *bmaE* genes were not detected in any of the UPEC isolates studied. VFs: Virulence factor-encoding genes.

**Table 2:** Serotype, *E. coli* phylogroup classification, and molecular characteristics of the hybrid uropathogenic/diarrheogenic *E. coli* (UPEC/DEC) isolates identified in this study.

Hybrid UPEC Identification	Serotype	Phylogroup	DEC ^a^ Markers				ExPEC Related Virulence Factor-Encoding Genes
			EPEC		EAEC		
			*escN*	*bfpB*	*aatA*	*aggR*	
34	O3:H2	A	-	-	+	+	*fimH, ecpA, papA/papC, iha, afaBC, hlyA, sat, irp2, traT, ompT*
69	O15:H6	D	-	-	+	+	*fimH, ecpA, papA/papC, iha, hlyA, sat, irp2, traT, ompT, picU*
85	O126:H10	B1	-	-	+	+	*fimH, ecpA, irp2, traT, ompT, sitA, iucD*
92	O145:H34	B2	+	-	-	-	*fimH, ecpA, ibeA, traT, ompT*
100	O90:H2	D	-	-	+	+	*fimH, ecpA, papC, traT, ompT, sitA*

^a^ Diarrheagenic *Escherichia coli* (DEC) pathotypes: EPEC: enteropathogenic *E. coli*; EAEC: enteroaggregative *E. coli*. None of the UPEC isolates studied harbored the *stx1* and/or *stx2* genes.

**Table 3:** Age, gender, type of urinary tract infection (UTI) and additional relevant clinical information from patients diagnosed with UTIs from which hybrid isolates of uropathogenic and diarrheagenic (UPEC/DEC) *E. coli* were obtained.

Hybrid UPEC/DEC Identification	Type of Hybrid ^a^	Patient Data:			
		Age (Years)	Gender ^b^	Type of UTI	Additional Information
34	UPEC/EAEC	11	F	Cystitis	Autistic Spectrum Disorder
69	UPEC/EAEC	22	F ^c^	Pyelonephritis	Fever
85	UPEC/EAEC	1	M	Cystitis	Prunc Belly Syndrome
92	UPEC/aEPEC	84	F	Cystitis	Diabetes melitus, hypertension and coronary artery disease
100	UPEC/EAEC	40	F	Cystitis	Renal transplantation and use of immunosuppressants

^a^ UPEC: uropathogenic *E. coli*, EAEC: enteroaggregative *E. coli*, and aEPEC: atypical enteropathogenic *E. coli*. ^b^ F: female and M: male. ^c^ Ten weeks of pregnancy.

**Table 4:** Antimicrobial resistance of the uropathogenic *E. coli* (UPEC) isolates studied.

Antimicrobial Drugs Tested	No. (%) of UPEC Isolates Classified in Each Category:		
	Susceptible	Intermediate ^a^	Resistant
β-lactams			
Ampicillin (AMP)	60 (53.6)	0	52 (46.4)
Piperacillin/Tazobactam (PPT)	109 (97.3)	3 (2.7)	0
Ampicillin/Sulbactam (ASB)	95 (84.8)	11 (9.8)	6 (5.4)
Amoxicillin/clavulanic acid (AMC)	93 (83)	15 (13.4)	4 (3.6)
Cephalothin (CFL)	51 (45.5)	40 (35.7)	21 (18.8)
Cefuroxime (CRX)	100 (89.3)	0	12 (10.7)
Ceftazidime (CAZ)	105 (93.8)	1 (0.9)	6 (5.4)
Cefotaxime (CTX)	101 (90.2)	0	11 (9.8)
Cefepime (CPM)	100 (89.3)	2 (1.8) ^a^	10 (8.9)
Ertapenem (ETP)	112 (100)	0 (0)	0 (0)
Meropenem (MER)	112 (100)	0 (0)	0 (0)
Aminoglycosides			
Gentamicin (GEN)	96 (85.7)	11 (9.8)	5 (4.5)
Amikacin (AMI)	105 (93.8)	7 (6.3)	0
Quinolones/Fluoroquinolones			
Nalidixic acid (NAL)	69 (61.6)	8 (7.1)	35 (31.3)
Norfloxacin (NOR)	87 (77.7)	2 (1.8)	23 (20.5)
Ciprofloxacin (CIP)	63 (56.3)	14 (12.5)	35 (31.3)
Folic acid metabolism inhibitors			
Trimethoprim/Sulfamethoxazole (SUT)	73 (65.2)	0	39 (34.8)
Nitrofuran			
Nitrofurantoin (NIT)	111 (99.1)	1 (0.9)	0
Phosphonic acid derivative			
Fosfomycin (FOS)	111 (99.1)	0	1 (0.9)

^a^ Or susceptible-dose dependent (SDD), if appropriate.

**Table 5:** Phenotypic and molecular features of the eleven Extended Spectrum Beta-Lactamase (ESBL)-producing uropathogenic *E. coli* (UPEC) isolates identified in this study.

UPEC Identification	Serotype	Phylogroup	Resistance Profile to β-Lactams:					β-Lactamase-Encoding Genes	
			Penicillins ^a,d^	Cephalosporins ^b,d^				Cephalosporinase ^c^	Additional Genes
				1st Gen.	2nd Gen.	3rd Gen.	4th Gen.		
10	ONT:HNM	*E. clades*	AMP (R)	CFL (R)	CRX (R)	CTX (R)	CPM (R)	*bla_CTX-M-8_*	*bla_TEM-1_*
20	ONT:HNM	A	AMP (R)	CFL (R)	CRX (R)	CAZ (R), CTX (R)	CPM (R)	*bla_CTX-M-15_*	*-*
30	ONT:HNM	A	AMP (R)	CFL (R)	CRX (R)	CAZ (I), CTX (R)	CPM (R)	*bla_CTX-M-15_*	*-*
55	OR:H51	B1	AMP (R), PPT (I), AMC (R)	CFL (R)	CRX (R)	CAZ (R), CTX (R)	CPM (R)	*bla_CTX-M-15_*, *bla_CMY-2_*	*bla_TEM-1_*
60	ONT:HNM	*E. clades*	AMP (R), ASB (I), AMC (R)	CFL (R)	CRX (R)	CAZ (R), CTX (R)	CPM (R)	*bla_CTX-M-15_*	*-*
85	O126:H10	B1	AMP (R), ASB (R), AMC (I)	CFL (R)	CRX (R)	CTX (R)	CPM (R)	*bla_CTX-M-8_*	*bla_TEM-1_*
94	OR:H4	B1	AMP (R), AMC (R)	CFL (R)	CRX (R)	CAZ (R), CTX (R)	CPM (R)	*bla_CTX-M-15_*, *bla_CMY-2_*	-
109	O101:HNM	A	AMP (R), ASB (I)	CFL (R)	CRX (R)	CTX (R)	CPM (R)	*bla_CTX-M-8_*	*bla_TEM-1_*
112	ONT:H4	A	AMP (R), ASB (I), AMC (I)	CFL (R)	CRX (R)	CAZ (R), CTX (R)	CPM (R)	*bla_CTX-M-15_*	*-*
114	O25:H4	B2	AMP (R), ASB (I), AMC (I)	CFL (R)	CRX (R)	CAZ (R), CTX (R)	CPM (R)	*bla_CTX-M-15_*	*bla_TEM-1_*
120	OR:H6	F	AMP (R)	CFL (R)	CRX (R)	CTX (R)	CPM (I)	*bla_CTX-M-15_*	*-*

^a^ Penicillins: ampicillin (AMP), Piperacillin/Tazobactam (PPT), ampicillin/sulbactam (ASB), amoxicillin/clavulanic acid (AMC). ^b^ Cephalosporins: cephalothin (CFL), cefuroxime (CRX), ceftazidime (CAZ), cefotaxime (CTX), and cefepime (CPM). ^c^ Cephalosporinase CTX-M-genes refer to groups. ^d^ Letters in parentheses indicate: resistant (R) or intermediate (I).

**Table 6:** Resistance profile of hybrid uropathogenic/diarrheogenic *E. coli* (UPEC/DEC) isolates identified in the present study.

Hybrid UPEC Identification	Type of Hybrid *E. coli* ^a^	ESBL-Producing UPEC	Classes of Antimicrobial Drugs ^b,c^:			
			β-Lactam	Aminoglycoside	Quinolone/Fluoroquinolone	Folic Acid Metabolism Inhibitor
34	UPEC/EAEC	-	AMP (R), ASB (R), AMC (I), CFL (I)	-	-	-
69	UPEC/EAEC	-	AMP (R), ASB (I), AMC (I)	-	-	-
85	UPEC/EAEC	+	AMP (R), ASB (R), AMC (I), CFL (R), CRX (R), CTX (R), CPM (R)	GEN (I)	NAL (I), CIP (R)	SUT (R)
92	UPEC/aEPEC	-	-	-	-	-
100	UPEC/EAEC	-	AMP (R), CFL (I)	GEN (I)	NAL (I)	SUT (R)

^a^ UPEC: uropathogenic *E. coli*, EAEC: enteroaggregative *E. coli*, and aEPEC: atypical enteropathogenic *E. coli*. ^b^ Classes of antimicrobial drugs: β-Lactams: ampicillin (AMP), ampicillin/sulbactam (ASB), amoxicillin/clavulanic acid (AMC), cephalothin (CFL), cefuroxime (CRX), cefotaxime (CTX), cefepime (CPM); Aminoglycoside: gentamicin (GEN); Quinolones: nalidixic acid (NAL), ciprofloxacin (CIP); Folic acid metabolism inhibitors: trimethoprim/sulfamethoxazole (SUT). ^c^ Letters in parentheses indicate: resistant (R) or intermediate (I).

## Data Availability

The raw data of this study will be made available by the authors, without reservation, to any qualified researcher.

## Supplementary Materials

The following supporting information can be downloaded at: https://www.mdpi.com/article/10.3390/microorganisms10030645/s1, Table S1: Primers used to detect virulence factor-encoding genes in the uropathogenic *E. coli* (UPEC) isolates studied [74,75,76,77,78,79,80,81,82,83,84,85,86,87,88,89,90,91]; Table S2: Primers used to detect virulence factor-encoding genes associated with the distinct diarrheagenic *E. coli* (DEC) pathotypes [92,93,94,95,96]; Table S3: Primers used to detect β-lactamase-encoding genes in the Extended Spectrum Beta-Lactamases (ESBL)-producing uropathogenic *E. coli* (UPEC) isolates identified in this study [97,98,99,100,101,102]; Table S4: Age, gender and clinical diagnosis of the outpatients with urinary tract infections included in this study; Table S5: Serotypes and *E. coli* phylogroups of the uropathogenic *E. coli* (UPEC) isolates studied; and Table S6: Resistance profile of the 31 uropathogenic *E. coli* (UPEC) isolates with multidrug-resistance (MDR) phenotype.
