# Supplementary material for: Characterization of Uropathogenic Escherichia coli Reveals Hybrid Isolates of Uropathogenic and Diarrheagenic (UPEC/DEC) E. coli
Source: Microorganisms. 2022 Mar 17;10(3):645. doi: 10.3390/microorganisms10030645 (PMC8950336; doi:10.3390/microorganisms10030645)
Supplement: Supplementary file 1 [file microorganisms-10-00645-s001.zip › microorganisms-1610341-supplementary.pdf]

SUPPLEMENTARY MATERIALS

Table S1. Primers used to detect virulence factor-encoding genes in the uropathogenic *E. coli* (UPEC) isolates studied.

Virulence factors investigated	Target gene	Primers sequence (5` → 3´)	Amplicon size (bp)	References
**Adhesins**				
Type 1 fimbriae	*fimH*	TGCAGAACGGATAAGCCGTGG<br>GCAGTCACCTGCCCTCCGGTA	508	[74]
P fimbriae	*papA*	ATGGCAGTGGTGTCTTTTGGTG<br>CGTCCCACCATACGTGCTCTTC	720	[74]
	papC	GACGGCTGTACTGCAGGGTGTGGCG<br>ATATCCTTTCTGCAGGGATGCAATA	328	[75]
Afa/Dr adhesins	*afaBC*	GCTGGGCAGCAAACTGATAACTCTC<br>CATCAAGCTGTTTGTTCGTCCGCCG	750	[75]
S/F1C fimbriae	*sfa/focDE*	CGGAGGAGTAATTACAAACCTGGCA<br>CTCCGGAGAACTGGGTGCATCTTAC	410	[75]
E. coli common pilus (ECP)	*ecpA*	GCAACAGCCAAAAAAGACACC<br>CCAGGTCGCGTCGAACT	477	[76]
IrgA homologue adhesin	*iha*	CAGGTCGGGGTTACCAAGT<br>CAAATGGCTCTCTTCCGTCAATGC	925	[77]
Heat-resistant agglutinin	*hra*	TCACTTGCAGACCAGCGTTTC<br>GTAACTCACACTGCTGTCACCT	537	[77]
M-agglutinin	*bmaE*	ATGGCGCTAACTTGCCATGCTG<br>AGGGGGACATATAGCCCCCTTC	507	[74]
**Invasin**				
Invasion of brain endothelium	*ibeA*	AGGCAGGTGTGCGCCGCGTAC<br>TGGTGCTCCGGCAAACCATGC	170	[74]

Table S1. *Continued*

Virulence factors investigated	Target gene	Primers sequence (5` → 3´)	Amplicon size (bp)	References
**Toxins**				
Hemolysin A	*hlyA*	AACAAGGATAAGCACTGTTCTGGCT<br>ACCATATAAGCGGTCATTCCCGTCA	1177	[74]
Cytotoxic necrotizing factor 1	*cnf1*	AAGATGGAGTTTCCTATGCAGGAG<br>CATTCAGAGTCCTGCCCTCATTATT	498	[79]
Cytolethal distending toxin	*cdt*	GAAARTAAATGGAAYAYAMATGTCCG<br>AATCWCCWRSAATCATCCAGTTA	466	[80]
Secreted autotransporter toxin	*sat*	TGCTGGCTCTGGAGGAAC<br>TTGAACATTCAGAGTACCGGG	667	[78]
vacuolating autotransporter toxin	*vat*	AACGGTTGGTGGCAACAATCC<br>AGCCCTGTAGAATGGCGAGTA	420	[81]
Uropathogenic specific protein	*usp*	ACATTCACGGCAAGCCTCAG<br>AGCGAGTTCCTGGTGAAAGC	448	[82]
Protein involved in colonization (Pic) from UPEC	*picU*	TCAGGCCGGTAAGAACAGCAAAAT<br>ACGGTAAGAGTGTGGATGGCGGAGTC	372	[83]
Temperature-sensitive hemagglutinin	*tsh*	ACTATTCTCTGCAGGAAGTC<br>CTTCCGATGTTCTGAACGT	824	[84]
Hemolysin F	*hlyF*	GGCGATTTAGGCATTCCGATACTC<br>ACGGGGTCGCTAGTTAAGGAG	599	[85]
**Iron acquisition**				
Salmochelin	*iroN*	AAGTCAAAGCAGGGGTTGCCCG<br>GACGCCGACATTAAGACGCAG	665	[86]
Yersiniabactin	*irp2*	AAGGATTCGCTGTTACCGGAC<br>TCGTCGGGCAGCGTTTCTTCT	264	[87]
Aerobactin	*iucD*	AAGTGTCGATTTTATTGGTGTA<br>CCATCCGATGTCAGTTTTCTG	760	[88]
Siderophore receptor IreA	*ireA*	ATTGCCGTGATGTGTTCTGC<br>CACGGATCACTTCAATGCGT	384	[78]
Periplasmic iron-binding protein	*sitA*	AGGGGGCACAACTGATTCTCG<br>TACCGGGCCGTTTTCTGTGC	608	[89]

Table S1. *Continued*

Virulence factors investigated	Target gene	Primers sequence (5` → 3´)	Amplicon size (bp)	References
**Protectins**				
Surface exclusion protein	*traT*	GGTGTGGTGCGATGAGCACAG<br>CACGGTTCAGCCATCCCTGAG	290	[74]
Outer membrane protease T	*ompT*	ATCTAGCCGAAGAAGGAGGC<br>CCCGGGTCATAGTGTTCATC	559	[90]
Increased serum survival	*iss*	ATCACATAGGATTCTGCCG<br>CAGCGGAGTATAGATGCCA	309	[91]
Group II capsule	*kpsMTII*	GCGCATTTGCTGATACTGTTG<br>CATCCAGACGATAAGCATGAGCA	272	[74]
Colicin V	*cvi/cvaC*	TCCAAGCGGACCCCTTATAG<br>CGCAGCATAGTTCCATGCT	597	[78]

Table S2. Primers used to detect virulence factor-encoding genes associated with the distinct diarrheagenic *E. coli* (DEC) pathotypes.

DEC pathotype[a]	Target gene	Primers sequence (5` → 3´)	Amplicon size (bp)	References
EPEC/EHEC	*escN*	CGACGACTATTGCAGAGT	499	[92]
		GCCTTATCTGCTTCAGGA		
Typical EPEC	*bfpB*	GACACCTCATTGCTGAAGTCG	910	[93]
		CCAGAACACCTCCGTTATGC		
STEC	*stx1/2*	GAGCGAAATAATTTATATGTG	518	[94]
		TGATGATGGCAATTCAGTAT		
EAEC	*aatA*	CTGGCGAAAGACTGTATCAT	630	[95]
		CAATGTATAGAAATCCGCTGTT		
	aggR[b]	GTATACACAAAAGAAGGAAGC	254	[96]
		ACAGAATCGTCAGCATCAGC		

[a]Diarrheagenic *E. coli* (DEC) pathotypes investigated: EPEC: enterophatogenic *E. coli*, STEC: shiga toxin-producing *E. coli*, and EAEC: enteroaggregative *E. coli*. EHEC (enterohemorrhagic *E. coli*) is a subgroup of the STEC pathotype that besides the shiga toxin-encoding genes (*stx1* and/or *stx2*), also harbor the LEE (locus of enterocyte effacement) region (*escN*$^+$).

[b]The *aggR* gene, encoding a transcriptional regulator, is used to differentiated typical (*aatA*$^+$/*aggR*$^+$) than atypical (*aatA*$^+$/*aggR*$^-$) EAEC isolates.

Table S3. Primers used to detect β-lactamase-encoding genes in the Extended Spectrum Beta-Lactamases (ESBL)-producing uropathogenic *E. coli* (UPEC) isolates identified in this study.

Target gene	Primers sequence (5` → 3´)	Amplicon size (bp)	References
bla_{SHV}	ATTTGTCGCTTCTTTACTCGC<br>TTTATGGCGTTACCTTTGACC	1051	[97]
bla_{TEM-1}	ATGAGTATTCAACATTTCCGTG<br>TTACCAATGCTTAATCAGTGAG	840	[98]
$bla_{CTX-M-2-group}$	AAATGTGCTGCTCCTTTCGTGAGC<br>AGGGTTCGTTGCAAGACAAGACTG	1112	[99]
$bla_{CTX-M-8-group}$	GCAAGCGCATTTTTGTTTTT<br>GACGACTTTCTGCCTTCTGC	900	[100]
$bla_{CTX-M-15-group}$	CACACGTGGAATTTAGGGACT<br>GCCGTCTAAGGCGATAAACA	499	[101]
bla_{CMY-2}	ATGATGAAAAAATCGTTATGCT<br>TTATTGCAGCTTTTCAAGAATGCG	1140	[102]

Table S4. Age, gender and clinical diagnosis of the outpatients with urinary tract infections included in this study.

**Age and Clinical diagnosis**	**No. of Patients (%)**		
	**Patients Gender:**		**Total**
	**Male (*n* = 11)**	**Female (*n* = 101)**	**(*n* = 112)**
**Age range**			
0-10	3 (27.3)	6 (5.9)	9 (8.0)
11-17	1 (9.1)	7 (6.9)	8 (7.1)
18-40	0	33 (32.7)	33 (29.5)
41-65	2 (18.2)	33 (32.7)	35 (31.3)
> 65	5 (45.4)	22 (21.8)	27 (24.1)
**Clinical diagnosis**			
Cystitis	8 (72.7)	79 (78.2)	87 (77.7)
Pyelonephritis	2 (18.2)	9 (8.9)	11 (9.8)
Asymptomatic bacteriuria (ABU)	1 (9.1)	10 (9.9)	11 (9.8)
No information available	0	3 (3.0)	3 (2.7)

Table S5. Serotypes and *E. coli* phylogroups of the uropathogenic *E. coli* (UPEC) isolates studied.

Serogroup[a]	H type[b]	Phylogroup	UPEC Identification[c]	No. of Isolates (%)
**O1**	H7	F	87	1 (0.9)
	H25	F	12	1 (0.9)
	HNM	D	53	1 (0.9)
**O2**	H7	B2	25, 80	2 (1.8)
**O3**	H2	A	34	1 (0.9)
**O4**	H4	B2	8, 68	2 (1.8)
**O6**	H1	B2	2, 24, 32, 71, 75, 76, 99, 102	8 (7.1)
	H31	B2	27, 45, 74, 88, 96	5 (4.5)
	HNM	B2	50	1 (0.9)
**O8**	H40	B1	62	1 (0.9)
**O15**	H2	G	6	1 (0.9)
	H6	D	69	1 (0.9)
**O16**	H5	B2	33	1 (0.9)
**O17**	H18	D	4	1 (0.9)
**O18**	H7	B2	5	1 (0.9)
	H31	B2	26, 35	2 (1.8)
**O20**	H9	C	15	1 (0.9)
**O21**	H12	A	7	1 (0.9)
	H21	B1	49	1 (0.9)
	HNM	B2	29	1 (0.9)
**O25**	H4	B2	18, 21, 28, 89, 90, 101, 114, 117	8 (7.1)
	H18	G	108	1 (0.9)
	HNM	A	118	1 (0.9)
**O55**	H8	G	48	1 (0.9)
**O75**	HNM	B2	23	1 (0.9)
**O81**	H14	B1	58	1 (0.9)
**O82**	H18	D	3	1 (0.9)
**O83**	H4	B2	73	1 (0.9)
	H33	B2	70	1 (0.9)
**O84**	H14	B1	13	1 (0.9)
**O86**	H2	D	22, 41	2 (1.8)
**O90**	H2	D	100	1 (0.9)
**O101**	HNM	A	109	1 (0.9)
**O103**	H7	B1	14	1 (0.9)
**O110**	H28	B1	107	1 (0.9)
**O126**	H10	B1	85	1 (0.9)
**O139**	H19	B1	81	1 (0,9)
**O145**	H34	B2	92	1 (0.9)
**O153**	H2	D	59	1 (0.9)
**O160**	H11	B1	52	1 (0.9)
**O164**	HNM	F	78	1 (0.9)
**O172**	H5	B2	31	1 (0.9)

Table S5. *Continued*

Serogroup[a]	H type[b]	Phylogroup	UPEC Identification[c]	No. of Isolates (%)
**ONT**	H1	B2	47	1 (0.9)
	H4	A	112	1 (0.9)
		B2	9, 116	2 (1.8)
		G	40	1 (0.9)
	H5	A	44	1 (0.9)
	H7	A	61	1 (0.9)
		B1	54	1 (0.9)
		B2	82, 97	2 (1.8)
	H8	E	110	1 (0.9)
	H9	C	72	1 (0.9)
	H14	D	115	1 (0.9)
	H18	B1	57	1 (0.9)
		D	38, 113, 119	3 (2.7)
	H25	B1	37	1 (0.9)
	H30	E	91	1 (0.9)
	HNM	A	17, 19, 20, 30, 36, 65, 79, 93	8 (7.1)
		B1	16	1 (0.9)
		B2	42	1 (0.9)
		F	63	1 (0.9)
		E. clades	10, 60	2 (1.8)
**OR**	H4	B1	94	1 (0.9)
		B2	39, 56	2 (1.8)
		D	111	1 (0.9)
	H6	F	120	1 (0.9)
	H10	B1	51	1 (0.9)
	H18	B2	46, 106	2 (1.8)
	H33	A	95	1 (0.9)
	H34	F	43	1 (0.9)
	H45	D	64	1 (0.9)
	H51	B1	55	1 (0.9)
	HNM	A	11, 98	2 (1.8)
		F	1, 103	2 (1.8)

[a]ONT: O-non-typeable and OR: rough (autoagglutinable) isolates
[b]HNM: non-motile UPEC isolates.
[c]Eight UPEC isolates (UPEC identification: 66, 67, 77, 83, 84, 86, 104 and 105) were not evaluated in the present study.

Table S6. Resistance profile of the 31 uropathogenic *E. coli* (UPEC) isolates with multidrug-resistance (MDR) phenotype.

**UPEC identification**[a]	**Classes of Antimicrobial Drugs**[b,c]**:**					
	**β-Lactam**	**Aminoglycoside**	**Quinolone/ Fluoroquinolone**	**Folic acid Metabolism inhibitor**	**Nitrofuran**	**Phosphonic acid derivative**
10	AMP (R), CFL (R), CRX (R), CTX (R), CPM (R)	-	NAL (R), NOR (R), CIP (R)	SUT (R)	-	-
15	AMP (R), ASB (R), AMC (I), CFL (I)	-	NAL (R), NOR (R), CIP (R)	SUT (R)	-	-
18	AMP (R)	GEN (R)	NAL (R), NOR (R), CIP (R)	SUT (R)	-	-
19	AMP (R), CFL (I)	-	NAL (R), NOR (R), CIP (R)	SUT (R)	-	-
20	AMP (R), CFL (R), CRX (R), CAZ (R), CTX (R), CPM (R)	-	NAL (R), NOR (R), CIP (R)	SUT (R)	-	-
21	CFL (I)	GEN (I), AMI (I)	CIP (I)	-	-	-
23	AMP (R), AMC (I), CFL (R), CPM (I)	AMI (I)	NAL (R), NOR (R), CIP (R)	-	-	-
26	AMP (R), ASB (R), AMC (I), CFL (R)	-	CIP (I)	SUT (R)	-	-
30	AMP (R), CFL (R), CRX (R), CAZ (I), CTX (R), CPM (R)	AMI (I)	NAL (R), NOR (R), CIP (R)	SUT (R)	-	-
37	AMP (R)	AMI (I)	NAL (R), CIP (I)	SUT (R)	-	-
43	AMP (R), AMC (R), CFL (R), CRX (R)	GEN (R)	NAL (R), NOR (R), CIP (R)	SUT (R)		-
48	AMP (R), CFL (I)	-	NAL (R), NOR (R), CIP (R)	SUT (R)	-	-
49	AMP (R), ASB (I), AMC (I), CFL (I)		CIP (I)	SUT (R)		-
55	AMP (R), PPT (I), AMC (R), CFL (R), CRX (R), CAZ (R), CTX (R), CPM (R)	-	NAL (R), NOR (R), CIP (R)	SUT (R)	-	FOS (R)
57	AMP (R), ASB (R), AMC (I), CFL (I)	GEN (R)	-	SUT (R)	-	-
60	AMP (R), ASB (I), AMC (R), CFL (R), CRX (R), CAZ (R), CTX (R), CPM (R)	-	NAL (R), NOR (R), CIP (R)	SUT (R)	-	-
71	CFL (I)	AMI (I)	NAL (I), CIP (R)	-	-	-
72	CFL (I)	-	NAL (R), CIP (R)	SUT (R)	-	-

Table S6. *Continued*

UPEC identification[a]	Classes of Antimicrobial Drugs[b,c]: β-Lactam	Aminoglycoside	Quinolone/ Fluoroquinolone	Folic acid Metabolism inhibitor	Nitrofuran	Phosphonic acid derivative
82	AMP (R), ASB (I), AMC (I), CFL (I)	-	NAL (R), CIP (R)	SUT (R)	-	-
85	AMP (R), ASB (R), AMC (I), CFL (R), CRX (R), CTX (R), CPM (R)	GEN (I)	NAL (I), CIP (R)	SUR (R)	-	-
89	AMP (R)	-	NAL (R), NOR (R), CIP (R)	SUT (R)	-	-
90	AMP (R), CFL (I)	GEN (I)	NAL (R), NOR (R), CIP (R)	-	-	
94	AMP (R), AMC (R), CFL (R), CRX (R), CAZ (R), CTX (R), CPM (R)	-	NAL (R), NOR (R), CIP (R)	SUT (R)	-	-
99	CFL (I)	GEN (I)	NAL (R), CIP (I)	-	-	-
100	AMP (R), CFL (I)	GEN (I)	NAL (I)	SUT (R)	-	-
101	AMP (R)	GEN (I)	NAL (R), NOR (R), CIP (R)	SUT (R)	-	-
109	AMP (R), ASB (I), CFL (R), CRX (R), CTX (R), CPM (R)	-	NAL (R), NOR (R), CIP (R)	SUT (R)	-	-
112	AMP (R), ASB (I), AMC (I), CFL (R), CRX (R), CAZ (R), CTX (R), CPM (R)	GEN (R)	NAL (R), NOR (R), CIP (R)	SUT (R)	NIT (I)	-
114	AMP (R), ASB (I), AMC (I), CFL (R), CRX (R), CAZ (R), CTX (R), CPM (R)	GEN (R)	NAL (R), NOR (R), CIP (R)	SUT (R)	-	-
119	AMP (R), CFL (R)	GEN (I)	-	SUT (R)	-	-
120	AMP (R), CFL (R), CRX (R), CTX (R), CPM (I)	-	NAL (R), NOR (R), CIP (R)	SUT (R)	-	-

[a]ESBL-producing UPEC isolates: 10, 20, 30, 55, 60, 85, 94, 109, 112, 114, and 120.

[b]Classes of antimicrobial drugs: **β-Lactams:** ampicillin (AMP), Piperacillin/Tazobactam (PPT), ampicillin/sulbactam (ASB), amoxicillin/clavulanic acid (AMC). cephalothin (CFL), cefuroxime (CRX), ceftazidime (CAZ), cefotaxime (CTX), cefepime (CPM); **Aminoglycoside:** gentamicin (GEN), amikacin (AMI); **Quinolones:** nalidixic acid (NAL), norfloxacin (NOR), ciprofloxacin (CIP); **Folic acid metabolism inhibitors:** trimethoprim/sulfamethoxazole (SUT); **Nitrofuran:** nitrofurantoin (NIT); and **Phosphonic acid derivative:** fosfomycin (FOS).

[c]Letters in parentheses indicate: resistant (R) or intermediate (I).
